# An Effective Near-Field to Far-Field Transformation with Planar Spiral Scanning for Flat Antennas under Test

**DOI:** 10.3390/s23167276

**Published:** 2023-08-19

**Authors:** Florindo Bevilacqua, Francesco D’Agostino, Flaminio Ferrara, Claudio Gennarelli, Rocco Guerriero, Massimo Migliozzi, Giovanni Riccio

**Affiliations:** 1 Department of Industrial Engineering, University of Salerno, Via Giovanni Paolo II, 84084 Fisciano, Italy; fbevilacqua@unisa.it (F.B.); fdagostino@unisa.it (F.D.); flferrara@unisa.it (F.F.); rguerriero@unisa.it (R.G.); mmigliozzi@unisa.it (M.M.); 2 Department of Information and Electrical Engineering and Applied Mathematics, University of Salerno, Via Giovanni Paolo II, 84084 Fisciano, Italy; griccio@unisa.it

**Keywords:** antennas measurements, non-redundant sampling representations, planar spiral scanning, near-to-far-field transformations

## Abstract

The goal of this article is to provide numerical and experimental assessments of an effective near-field to far-field transformation (NF–FF T) technique with planar spiral scanning for flat antennas under test (AUTs), which requires a non-redundant, i.e., minimum, number of NF measurements. This technique has its roots in the theory of non-redundant sampling representations of electromagnetic fields and was devised by suitably applying the unified theory of spiral scans for non-volumetric antennas to the case in which the considered AUT is modeled by a circular disk having its radius equal to half of the AUT’s maximum dimension. It makes use of a 2D optimal sampling interpolation (OSI) formula to accurately determine the massive amount of NF data required by the classical plane-rectangular NF–FF T technique from the non-redundant data gathered along the spiral. It must be emphasized that, when considering flat AUTs, the developed transformation allows one to further and significantly save measurement time as compared to that required by the previously developed NF–FF T techniques with planar spiral scans based on a quasi-planar antenna modeling, because the number of turns of the spiral and that of NF data to be acquired depend somewhat on the area of the modeling surface. The reported numerical simulations assess the accuracy of the proposed NF–FF T technique, whereas the experimental tests prove its practical feasibility.

## 1. Introduction

Antenna measurements concerning the pattern representation of a source under test need to reduce the time devoted to the data collection for the purposes of increasing the number of tests in the available time slot. This target can be accomplished in the far-field (FF) region as well as in the near-field (NF) volume surrounding the antenna under test (AUT) by adopting strategies which allow (a) a reduction in the amount of data that needs to be acquired and (b) the adoption of non-conventional scanning techniques not requiring step-by-step acquisition. Requirement (a) can be satisfied by resorting to the theoretical results concerning the non-redundant sampling representation of the electromagnetic (EM) field [1] radiated by antennas, whereas on-the-fly data acquisition permitted by the continuous movements of positioners can be used to fulfill requirement (b).

A non-redundant sampling representation of the EM field radiated by the AUT permits one to determine the field value at any observation point on surfaces in the NF or FF region by employing the minimum number of data [1] available on the same surface. Such data can be properly expended in optimal sampling interpolation (OSI) algorithms to recover the needed field values. Such a procedure is extremely convenient when dealing with NF data to be used in NF–FF transformation (NF–FF T) techniques [2,3,4,5,6,7,8,9,10,11,12]; for instance, in non-redundant NF–FF T techniques with plane-rectangular, plane-polar, and bi-polar scanning [12], efficient OSI algorithms have been proposed to precisely recover the NF data necessary for the standard plane-rectangular NF–FF T technique [13,14].

Innovative and non-conventional NF spiral scans based on Rahmat-Samii’s idea [15] utilize on-the-fly data gathering by adopting the continuous and synchronized movements of the positioners driving AUT and measuring probes. In particular, NF–FF Ts using sampling points on a planar spiral have been developed to reduce the acquisition time [16,17,18]. This reduction is accomplished accounting for the theory of non-redundant sampling representations [1] and for the unified theory of spiral scanning for volumetric [19] and non-volumetric AUTs [20]. In fact, the number of spiral turns required to cover a given scanning area decreases since its step must satisfy the non-redundant sampling rule associated with a radial line, and the number of sampling points on the resulting spiral is the minimum one. It must be also emphasized that the percentage of reduction depends on the area of the closing surface Σ adopted to model the antenna [1], f.i., by assuming as standard AUT modeling the spherical surface with radius equal to the AUT half-dimension, the numbers of spiral turns and sampling points decrease if an oblate spheroid, which is enclosed in the above spherical surface, is used as AUT modeling [17,18]. On the other hand, a two-bowl (i.e., a surface formed by two circular bowls with the same aperture and lateral bends which may differ) model can represent the best choice in the case of quasi-planar AUTs [18], as it generally permits a better fitting of their shape.

The following question now arises: is it possible to lower the number of spiral turns and the number of corresponding sample points in the case of a flat conventional or non-conventional [21,22] AUT? The answer is described in the following sections of this article, which are organized as follows. The theoretical analysis is given in Section 2 by employing a disk with a radius equal to half the AUT’s dimensions as the AUT model. Section 3 is devoted to numerical tests and comparisons with measured data. Conclusions are collected in Section 4.

## 2. OSI Representation on a Plane from Spiral Samples

The proposed acquisition strategy is introduced in this section, as well as the formulation of the innovative disk modeling for flat antennas and the related OSI formulas.

**Figure 1:**
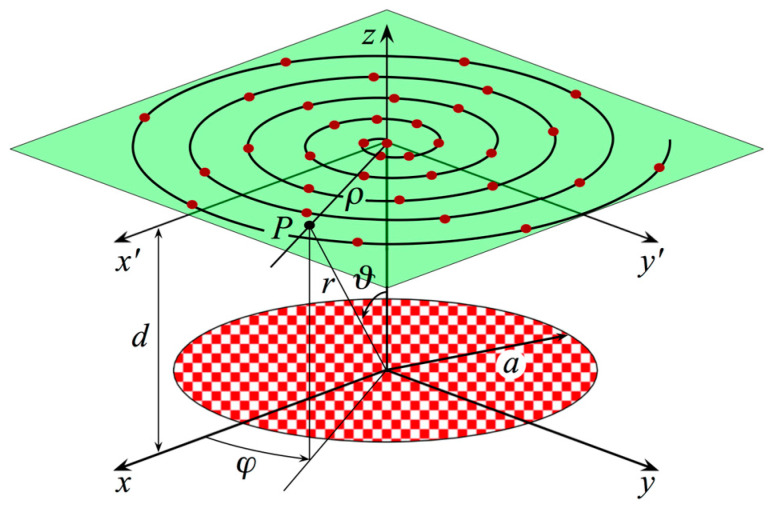
Geometry relevant to the planar spiral scan for a flat antenna.

It is used to perform the characterization of a flat AUT by means of a planar spiral NF facility, which acquires the NF voltages over a plane set at distance *d* from it. Furthermore, it is assumed to adopt an electrically small, first-order probe, namely a probe whose FF pattern has a first-order azimuthal dependence, to carry out the acquisition of the required data. Such an assumption will be properly exploited in the following. Moreover, it is also convenient to introduce a spherical and a Cartesian reference coordinate system, (r,ϑ,φ) and (*x*, *y*, *z*), respectively, both with their centers at *O*, to specify an observation point *P*. Such a point can be identified on the scanning plane using the plane-polar coordinates (ρ,φ), as depicted in Figure 1.

**Figure 2:**
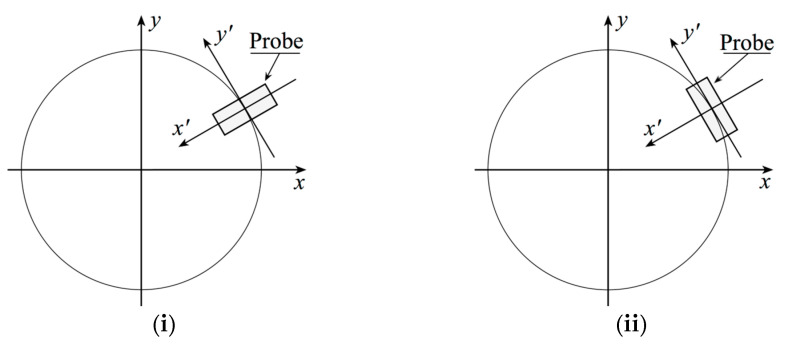
(**i**) Measurement of $V_{\varphi }$; (**ii**) measurement of $V_{\rho }$.

It is interesting to note that the voltage revealed at the output terminals of the considered scanning probe shows the same effective spatial bandwidth as the field [23]. Such a property allows one to apply the results presented in [1] also to the acquired voltage. Accordingly, a convenient representation of the voltage over any curve Γ of the plane can be properly formulated by describing such a curve by an opportune parameter *ξ* and referring to the “reduced voltage”.

(1) $$\tilde{V}(\xi )=\;V(\xi )\;e^{j\gamma (\xi )}$$

wherein γ(ξ) denotes an opportune phase function to be calculated and the voltage *V* is equal to $V_{\varphi }(\xi )$ or $V_{\rho }(\xi )$ depending on whether the probe is in the nominal orientation or rotated by π/2 (see Figure 2). According to [1], *V* is a function spatially quasi bandlimited to $W_{\xi }$. It can be very well approximated by a function bandlimited to $\chi^{\prime }W_{\xi }$, by properly setting the excess bandwidth factor $\chi^{\prime }$, which makes it possible to control the aliasing error. It should be noted that a $\chi^{\prime }$-value slightly greater than one can be suitably used when dealing with the characterization of electrically large AUTs [1].

By following the reasoning derived in [20], it is possible to reconstruct the voltage distribution efficiently and accurately over the acquisition plane from a reduced set of NF data gathered on the spiral by applying a 2D OSI formula. The OSI scheme can be derived [20] by enforcing that the spiral step has the sampling spacing necessary to perform the interpolation on a radial line. After that, a suitable non-redundant sampling representation over this spiral is formulated.

As shown in [1], the parameter to be employed to provide the optimal representation along radial lines, the associated phase function, and spatial bandwidth are given by:

(2) $$\;\xi =\left(\pi /l^{\prime }\right)\left[R_{1}-R_{2}+s_{1}^{\prime }+s_{2}^{\prime }\right]$$

(3) $$\;\gamma =\left(\pi /\lambda \right)\left[R_{1}+R_{2}+s_{1}^{\prime }-s_{2}^{\prime }\right]$$

(4) $$W_{\xi }=l^{\prime }/\lambda$$

where $l^{\prime }$ denotes the length of $\Gamma^{\prime }$ (intersection curve between the meridian plane at the observation point *P* and Σ), λ is the wavelength, $R_{1}$ and $R_{\;2}$ are the distances from *P* to the tangency points $P_{1}$ and $P_{\;2}$ on such a curve, and $s_{1}^{\prime }$ and $s_{2}^{\prime }$ are their curvilinear abscissae, respectively.

The relations (2)–(4) are general and, to find their explicit expressions, it is necessary to specify the modeling surface Σ containing the AUT. It is important to highlight that the number $N_{S}$ of samples at the Nyquist rate on an arbitrary closed surface (even unbounded), which encircles the antenna, can be expressed as

(5) $$N_{S}≅\;\;\;\frac{\mathrm{area}\;\mathrm{of}\;\Sigma }{{\left(\lambda /2\right)}^{2}}$$

Therefore, it is evident that, by properly choosing the antenna modeling, it is possible to minimize the overall amount of needed NF data. In fact, a convenient approach to cut the number of required NF samples is to minimize the area of the surface which models the antenna by choosing a geometry which fits very well to the source shape and so reducing the volumetric redundancy.

As a result, when considering flat antennas, the modeling surface which corresponds to the smallest area is that of a disk with a radius *a* equal to half their maximum size, as it is capable of fitting to their geometry as much as possible. It should be noted that the NF data reduction achievable by using such a modeling approach is much greater than that resulting from the usage of the previously derived models for quasi-planar antennas (two-bowl or oblate spheroid), which involve a residual volumetric redundancy.

**Figure 3:**
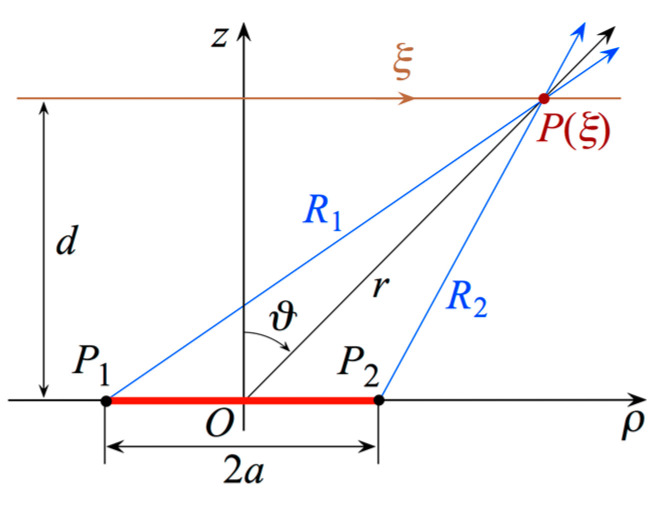
Geometry relevant to a radial line.

By shaping the AUT with a disk of radius *a,* since (see Figure 3) $l^{\prime }=4\;a$, $s_{1}^{\prime }=-a$, and $s_{2}^{\prime }=a$, the relations from (2) to (4) can be particularized as:

(6) $$\xi =\left(\pi /4a\right)\;\left(R_{1}-R_{2}\right)$$

(7) $$\gamma =\left(\pi \;/\lambda \right)\;\left(R_{1}+R_{2}-2a\right)$$

(8) $$W_{\xi }=4a/\lambda$$

**Figure 4:**
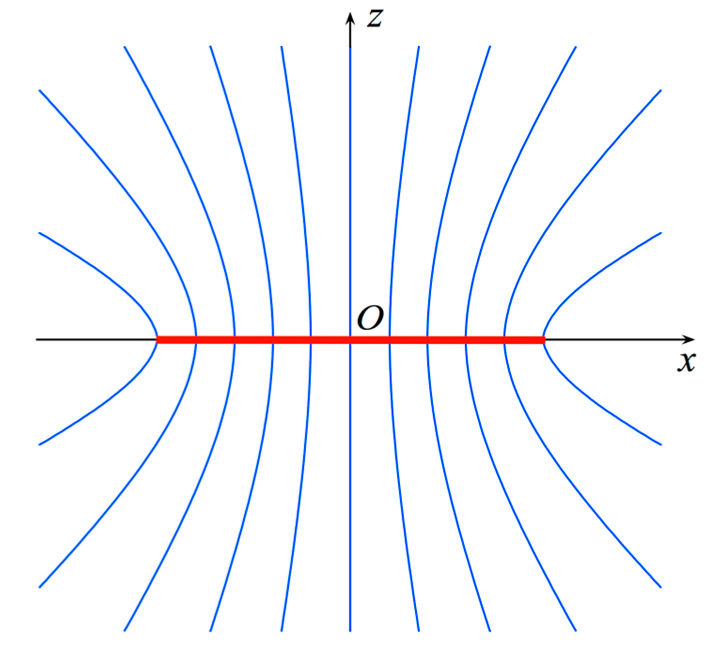
Curves at *ξ* = constant.

As shown in [20], the spiral lying on the scanning plane can be achieved as a projection through the curves at *ξ* = constant (Figure 4) of a spiral which wraps with a proper step the disk modeling the source.

This step must coincide with the sampling spacing necessary to carry out the interpolation along a radial line, namely $\Delta \xi \;\;=2\pi /\left(2N^{\prime \prime }+1\right)$, where $N^{\prime \prime }=\mathrm{Int}\left(\chi N^{\prime }+1\right)$, $N^{\prime }=\mathrm{Int}\left(\chi^{\prime }W_{\xi }+1\right)$, Int (*x*) denotes the integer part of *x*, and χ>1 is the oversampling factor to be used for controlling the truncation error [1]. Therefore, the equations describing the acquisition spiral are as follows:

(9) $$\left\{\begin{array}{l} x=\rho (\xi )\cos\phi \\ y=\rho (\xi )\sin\phi \\ z=d \end{array}\right.$$

where *ϕ* denotes an angular parameter that allows one to describe the spiral, ρ(ξ)=d tanθ(ξ), and *ξ* = *k ϕ*. As two successive intersections at *Q* (*ϕ*) and *Q* (*ϕ* + 2π) of the considered radial line with the spiral determine its step, $k\;\;=1/\left(2N^{\prime \prime }+1\right)$ [20]. In addition, it is highlighted that the spiral angle *θ*, at variance with the zenithal angle *ϑ*, can also be negative. Furthermore, it is pointed out that the parameter *ϕ* is always continuous, whereas the azimuthal angle *φ* shows a discontinuity jump of π at the pole.

The unified theory of spiral scanning [20] is now exploited to develop the non-redundant representation on the spiral, namely the optimal parameter *η* and the related phase function *ψ*. In greater detail, the parameter *η* for representing the spiral path must be equal to $2\pi /(\lambda \;W_{\eta })$ times the curvilinear abscissa of the projection point, which lies on the spiral which wraps around the disk surface Σ. Furthermore, the associated phase function *ψ* needs to be the same as that (*γ*) derived for a radial line. As concerns the spatial bandwidth $W_{\eta }$, it can be suitably calculated by ensuring that the parameter *η* covers a 2π range when drawing the entire (closed) projecting spiral. Accordingly, $W_{\eta }$ is 2/λ times the length of the spiral that wraps around the disk from pole to pole [20].

In consideration of the aforementioned results, a fast and accurate way to reconstruct the voltage at the point *P,* on the radial line at *φ*, is to employ the following OSI formula [17,20]

(10) $$V\left(\xi (\rho ),\varphi \right)=e^{-j\gamma (\xi )}\sum_{n=n_{0}-q+1}^{n_{0}+q}\tilde{V}\left(\xi_{n}\right)OS\left(\xi ,\xi_{n},N,N^{\prime \prime },\bar{\xi }\right)$$

where 2*q* is the number of retained intermediate reduced voltage samples $\tilde{V}(\xi_{n})$, i.e., those in correspondence with the points of intersection of the acquisition spiral with the radial line passing through *P*, $\bar{\xi }=q\Delta \xi$, $N=N^{\prime \prime }\;-N^{\prime }$, and $n_{0}=\mathrm{Int}\left[(\xi -\xi_{0})/\Delta \xi \right]\;$,

(11) $$\xi_{n}=k\varphi +n\Delta \;\xi =\xi_{0}+n\Delta \;\xi$$

are the sampling points. In Equation (10),

(12) $$OS\left(\xi ,\xi_{n},N,N^{\prime \prime },\bar{\xi }\right)={\mathrm{TS}}_{N}\left(\xi -\xi_{n},\bar{\xi }\right)D_{N^{\prime \prime }\;}\left(\xi -\xi_{n}\right)\;\;$$

is the OSI kernel function [1,20], wherein

(13) $$TS_{N}\left(\xi ,\bar{\xi }\right)=\frac{T_{N}\left[2\cos^{2}\left(\xi /2\right)/\cos^{2}\left(\bar{\xi }/2\right)-1\right]}{T_{N}\left[2/\cos^{2}\left(\bar{\xi }/2\right)-1\right]}$$

is the Tschebyscheff sampling function, with $T_{N}(\xi )$ being the *N* degree Tschebyscheff polynomial, and

(14) $$D_{N^{\prime \prime }\;}\left(\xi \right)=\frac{\sin\left[(2N^{\prime \prime }+1)\xi /2\right]}{\left(2N^{\prime \prime }+1)\;\sin(\xi /2\right)}$$

is the Dirichlet function.

By properly taking into account the non-redundant representation on the scanning spiral, is possible to determine [17,20] the intermediate reduced voltage samples $\tilde{V}(\xi_{n})$ from those collected on the spiral through the OSI expansion:

(15) $$\tilde{V}\left(\eta (\xi_{n})\right)=\sum_{m=m_{0}-p+1}^{m_{0}+p}\tilde{V}\left(\eta_{m}\right)OS\left(\eta (\xi_{n}),\eta_{m},M,M^{\prime \prime }\;,\bar{\eta }\right)$$

where $m_{0}=\mathrm{Int}\left[\eta (\xi_{n})/\Delta \eta \right]\;$, 2*p* represents the number of considered NF samples on the spiral, $\bar{\eta }=p\Delta \eta$, $M^{\prime \prime }=\mathrm{Int}\left(\chi M^{\prime }+1\right)$, $M=M^{\prime \prime }\;-M^{\prime }$, $M^{\prime }=\mathrm{Int}\left(\chi^{\prime }W_{\eta }+1\right)$,

(16) $$\eta_{m}=m\;\Delta \eta ;\;\;\Delta \eta =2\;\pi \;/\left(2M^{\prime \prime }+1\right)$$

and the meaning of the other symbols is fairly similar to those ones in (10).

It is noteworthy that when applying the expansion (15) to recover the intermediate samples near to the pole ϑ=0, even small changes in *η* entail large changes in the angular parameter *ϕ*. As a consequence, the bandwidth excess factor $\chi^{\prime }$ to be used in (15) must be locally suitably increased to prevent the increase in the band-limitation error in this zone impairing the accuracy and the quality of the reconstruction.

The 2D OSI expansion, allowing the evaluation of the voltage distribution on the plane from the non-redundant samples gathered on the spiral, is finally obtained by matching the 1D expansions (10) and (15). It is conveniently exploited to recover the voltage value of $V_{\varphi }$ and $V_{\rho }$ at the sampling positions of the regular Cartesian grid required for executing the NF–FF T [14]. Unfortunately, the relations in [14], accounting for the effects of the probe, require the entry of the voltages $V_{x}$ and $V_{y}$ in order to be applied. This means that it is necessary to perform the co-rotation of the probe during the measurement stage, such that its axes are kept parallel to those of the AUT. However, this “hardware” co-rotation can be avoided by using, as already mentioned, a probe radiating a FF pattern showing only an azimuthal dependence of the first-order as, e.g., it occurs with a very close approximation for an open-ended rectangular waveguide excited by a ${\mathrm{TE}}_{10}$ mode [24]. As a matter of fact, in this case, the voltages $V_{x}$ and $V_{y}$, which would be measured by the probe and the rotated one with co-rotation, can be calculated from the measured non-co-rotated ones, $V_{\rho }$ and $V_{\varphi }$, by simply applying the following relations:

(17) $$V_{y}=V_{\varphi }\;\cos\;\varphi -V_{\rho }\sin\;\varphi ;\;V_{x}=V_{\varphi }\sin\;\varphi +V_{\rho }\cos\;\varphi$$

## 3. Results

An extensive number of numerical simulations and experimental tests, performed at the Antenna Characterization Lab of the University of Salerno (UNISA), were carried out to thoroughly assess the precision and reliability of the developed non-redundant spiral NF–FF T for flat antennas.

### 3.1. Numerical Tests Results

**Figure 5:**
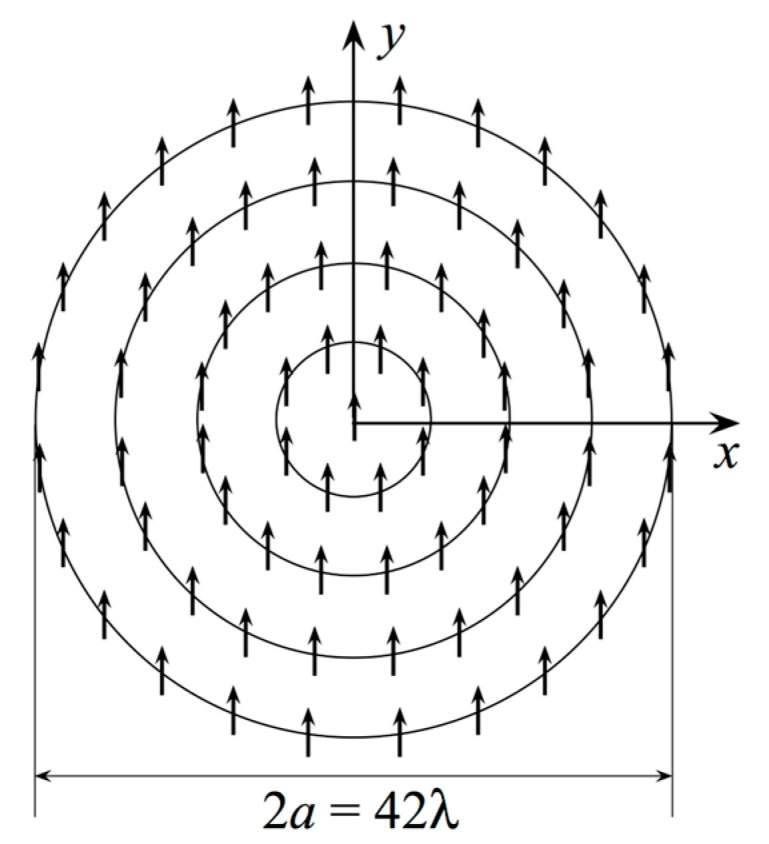
Geometry relevant to the simulated uniform circular planar array. The arrows represent the array elements polarized along the y-axis.

We show, in this section, numerical results that prove the efficacy of the developed NF–FF T technique when adopting a planar spiral scan. The considered AUT is a uniform circular planar array with radius *a* = 21.0 λ. The array was placed in the *xy* plane of the used reference system, as illustrated in Figure 1. The elements are elementary Huygens sources linearly polarized along the *y*-axis. They are symmetrically located with respect to the axes and are spaced azimuthally at approximately 0.7 λ intervals along the circumferences, which are radially spaced at 0.7 λ intervals (see Figure 5). Furthermore, they were fed in such a way that the AUT showed radiating characteristics similar to a Tschebyscheff array, with a first side lobe level of −40 dB. According to the proposed sampling representation, the AUT is assumed to be enclosed within a disk with an identical radius *a*. The NF samples of $V_{\varphi }$ and $V_{\rho }$ were numerically evaluated as collected using an open-ended WR-90 rectangular waveguide on a spiral, which covers a circle with radius ≈ 71 λ on a plane *d* = 8.0 λ away from the antenna’s center.

**Figure 6:**
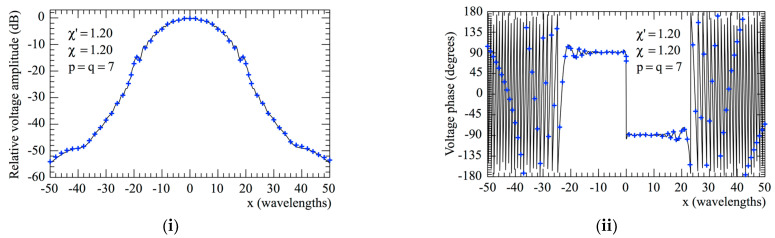
$V_{\varphi }$ on the radial line at *φ* = 0. –––––, reference; ++++, evaluated from the planar spiral NF measurements. (**i**) Amplitude; (**ii**) phase.

**Figure 7:**
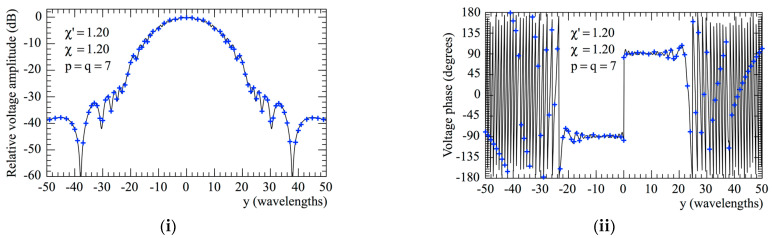
$V_{\rho }$ on the radial line at φ=π/2. –––––, reference; ++++, evaluated from the planar spiral NF measurements. (**i**) Amplitude; (**ii**) phase.

**Figure 8:**
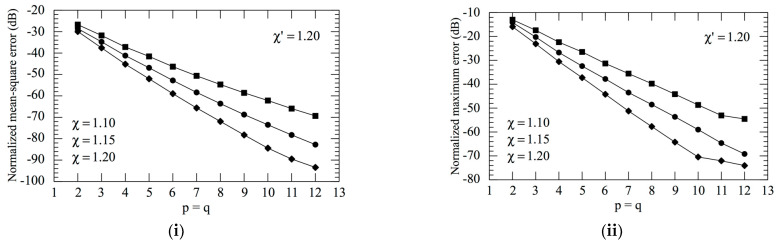
Errors in the evaluation of $V_{\rho }$. (**i**) Mean-square error; (**ii**) maximum error. Squares refer to χ = 1.10, circles to χ = 1.15 and rhombus to χ = 1.20.

The accuracy of the 2D OSI scheme, obtained by combining Formulas (10) and (15), can be qualitatively and quantitatively assessed. From the qualitative point of view, Figure 6 and Figure 7 show the comparisons of the reconstructed amplitudes and phases with the exact ones relevant to $V_{\varphi }$ and $V_{\rho }$ along the radial lines at *φ* = 0 and φ=π/2, respectively. It is evident as the reconstructions, both for the amplitudes and phases, match the references very well. It must be underlined that these successful recoveries were achieved by using an excess bandwidth factor $\chi^{\prime }=1.20$, which ensures a negligible band-limitation error [12], and *p* = *q* = 7, together with an oversampling factor *χ* = 1.20 to control the truncation one. Moreover, to prevent an increase in the band-limitation error in close proximity to the pole, the $\chi^{\prime }$ value employed in the interpolation on the spiral was locally increased in such a way that in the zone of the spiral specified by the 32 samples near the pole, the sample spacing was reduced by a factor of 11. Thus, the total number of NF samples on the spiral was 11,959, inclusive of the 320 “extra samples” at a reduced spacing around the pole. The discontinuity jump of 180° in the phase of the voltages $V_{\varphi }$ and $V_{\rho }$ in correspondence with the polar singularity, due to the change in the sign of $V_{\varphi }$ and $V_{\rho }$ at the pole (see Figure 2), is noteworthy. It is also interesting to provide quantitative proofs of the precision of the 2D OSI formula. To this end, the values of $V_{\rho }$, calculated through the 2D OSI formula at the points of a close lattice in the central zone of the scan plane (to ensure the availability of the guard samples), were compared with the exact values. This comparison was carried out by choosing $\chi^{\prime }=1.20$ and different values of *χ*, *p,* and *q*. The resulting mean-square and maximum reconstruction errors, normalized to the maximum value of the voltage on the plane, are shown in Figure 8. It is evident that, by increasing the values of *χ*, *p,* and *q*, the reconstruction errors decrease. This provides a criterion to select the appropriate values of the OSI parameters, once the tolerable level of reconstruction error has been fixed.

**Figure 9:**
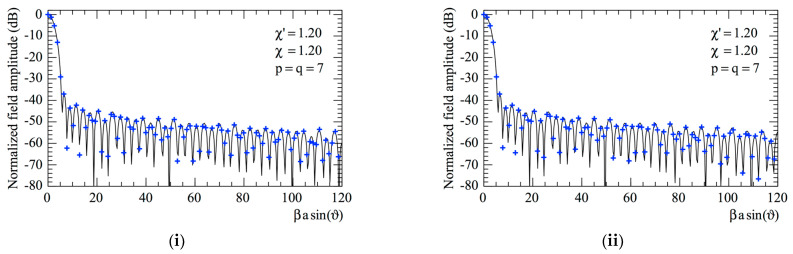
Far-field patterns. –––––, exact; ++++, reconstructed from the non-redundant samples. (**i**) E-plane; (**ii**) H-plane.

Finally, to evaluate the total efficacy of the proposed NF–FF T technique, the 2D OSI formula was used to precisely evaluate the voltages $V_{\varphi }$ and $V_{\rho }$ at the points of the Cartesian grid required to execute the standard Leach and Paris NF–FF T technique [14]. Then, it is possible to exploit (17) to determine the corresponding needed values of $V_{x}$ and $V_{y}$, since, as previously stressed, the utilized probe radiates, with a very good approximation [24], a far field with a first-order azimuthal dependence. The E-plane and H-plane radiation patterns obtained from the 40,401 NF data reconstructed on the 0.5 λ-spaced square grid with a side length of 100 λ, inscribed in the scanning circle, are compared to the exact patterns in Figure 9. As can be noted, the agreement of the thus obtained FF patterns with the exact ones is very good also in the far out sidelobes region.

Before concluding the numerical section, it is interesting to highlight the data reduction obtained by employing the disk modeling of the AUT. To this end, Table 1 reports the comparison of the NF samples required in the various approaches used to cover the scanning area of 100 λ×100 λ. Interestingly, the proposed approach entails a significant reduction in the number of NF samples as compared to the Leach and Paris NF–FF T technique [14], as well as with respect to the classical plane-polar NF–FF T technique by Rahmat-Samii et al. [25,26,27]. Also, the amount of data in the devised approach compares favorably with respect to the number of samples necessary to execute the NF–FF Ts with planar spiral scanning when adopting an oblate spheroid [17,18] or a two-bowl [18] model of the AUT. In particular, the oblate spheroid model considered in the comparison has its semi-minor and semi-major axes equal to 21 λ and 6 λ, respectively, whilst the double-bowl model has its aperture radius equal to 21 λ and the radii of the lower and upper lateral bends are both equal to 2.5 λ. Finally, the use of the disk as AUT modeling is computationally much more simple than these other models, since the expressions of the parameters involved in the representation are simpler. This highlights the efficiency and effectiveness of the proposed approach in reducing the amount of needed data, while keeping performance comparable to previous methods.

### 3.2. Experimental Tests Results

**Figure 10:**
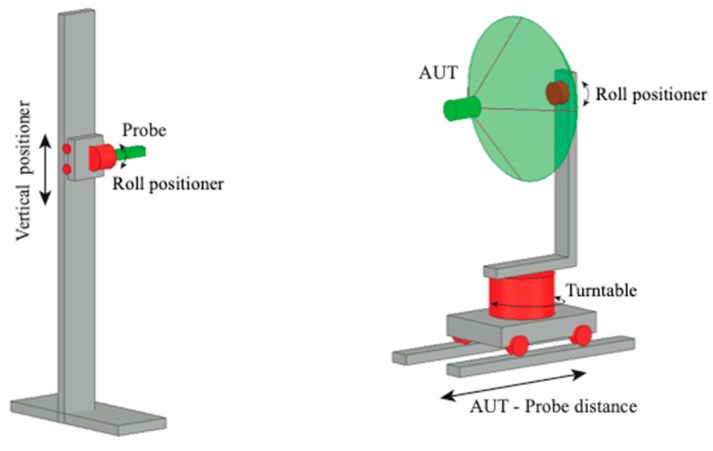
Pictorial illustration of the “versatile” NF setup existing at the UNISA Antenna Characterization Laboratory.

Some results of experimental tests which assess the validity of the here-proposed NF–FF T technique with a planar spiral scan are presented in the following. The experimental tests were carried out at the Laboratory of Antenna Characterization at the University of Salerno. This laboratory is supplied with an anechoic chamber with dimensions of 8 m × 5 m × 4 m, covered by pyramidal absorbers, which guarantee a reflectivity level lower than −40 dB. It is equipped with a “versatile” NF acquisition setup that offers the capability of performing spherical, cylindrical, plane-polar, and spiral scans, including the proposed scanning along a planar spiral. To perform the plane-polar scanning and, hence, the planar spiral scanning, the probe is attached to a vertical slide and the AUT is placed on a turntable with its axis perpendicular to the vertical slide. To expand the capabilities of the plane-polar NF facility, an additional rotary table is located between this positioner and the probe. Accordingly, this setup makes it possible to acquire the NF data that would be typically measured using plane-rectangular NF setups. Furthermore, this additional rotating table permits the measurement of the NF data that would be gathered in a planar spiral NF setup with hardware co-rotation, where the axes of the probe and the AUT are kept parallel throughout the acquisition process. A pictorial illustration of the acquisition setup is shown in Figure 10. A vector network analyzer is used to accomplish the measurement of amplitude and phase of the voltages collected using the employed probe, an open-ended WR-90 rectangular waveguide. As previously observed, this choice allows the software to perform co-rotation of the acquired voltage values by using (17).

**Figure 11:**
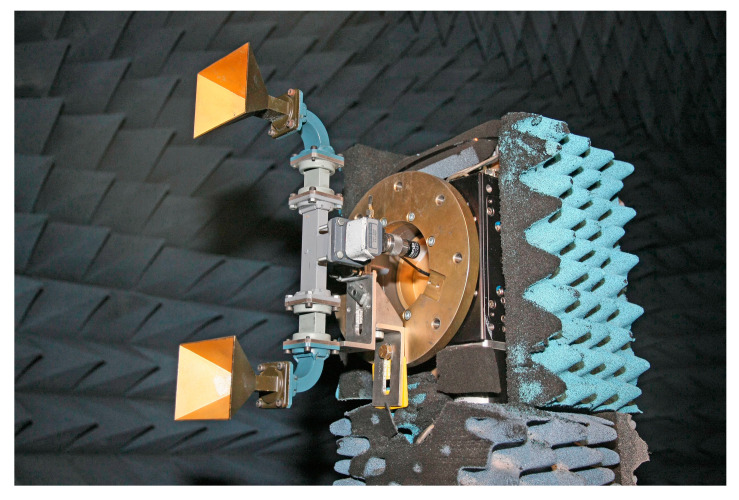
Photo of the considered AUT, taken before covering its support with the absorbers.

The AUT, a monopulse antenna in the E-plane working at 10 GHz in the sum mode, is realized by assembling two pyramid-shaped horns, fed by a hybrid Tee. The aperture of each horn, placed on the plane *xy*, has dimensions of 8.9 cm × 6.8 cm, and the aperture centers are 26.5 cm away (see Figure 11). Moreover, the AUT is oriented in such a way that the larger sides of the horn apertures are parallel to the *x* axis when φ=0.

According to the derived representation, this source is properly modeled by considering a disk with diameter 2*a* = 37.2 cm. The distance *d* between the measurement plane and the probe is 16.5 cm, and the samples of the probe voltages $V_{\rho }$ and $V_{\varphi }$ are acquired along a spiral which covers a circle with a radius of 114 cm. As regards the choice of the distance *d*, it should be sufficiently small to reduce the error due to the measurement area truncation for a fixed size of the scanning zone. It can be easily realized that, since the scanning plane must be external to the modeling surface Σ, the disk modeling allows one to reduce the measurement plane distance with respect to the other AUT models. However, such a distance cannot be reduced beyond certain limits in order to avoid a non-negligible mutual coupling between the probe and the AUT and to acquire the NF samples in the radiating NF region, where the evanescent waves of the AUT field are negligible. The sampling positions on the spiral were determined in accordance with the derived non-redundant sampling representation by choosing χ=1.25 and a $\chi^{\prime }$ value equal to 1.30, save for the interpolation along the spiral in the zone specified by the 24 samples near to the pole, where it was locally augmented such that the sample spacing was reduced by a factor of exactly 9. Thus, the total number of the NF measurements on the spiral was 1665, inclusive of the 192 “extra samples” at reduced spacing close to the pole.

**Figure 12:**
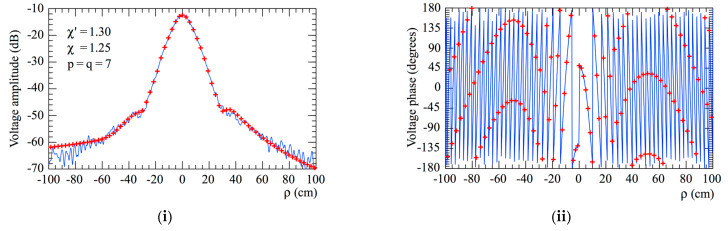
$V_{\varphi }$ on the radial line at *φ* = 0. –––––, reference; ++++, obtained from the planar spiral NF measurements. (**i**) Amplitude; (**ii**) phase.

**Figure 13:**
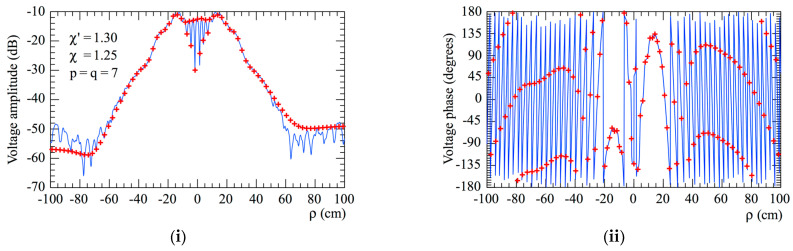
$V_{\rho }$ on the radial line at φ=π/2. –––––, reference; ++++, obtained from the planar spiral NF measurements. (**i**) Amplitude; (**ii**) phase.

**Figure 14:**
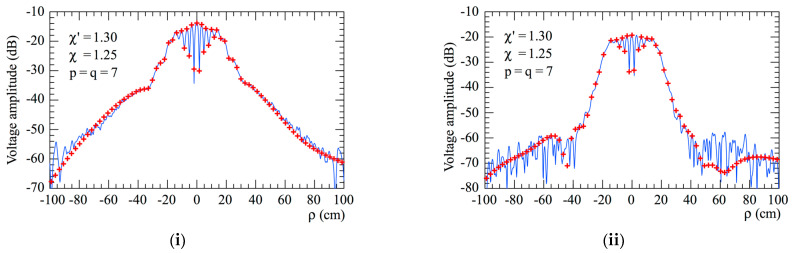
Voltage amplitudes on the radial line at φ=π/6. –––––, reference; ++++, obtained from the planar spiral NF samples. (**i**) Voltage $V_{\varphi }$; (**ii**) voltage $V_{\rho }$.

To assess the accuracy of the 2D OSI formula, the magnitudes and phases of the reconstructed voltages $V_{\varphi }$ and $V_{\rho }$, along the radial lines at φ=0 and φ=π/2, respectively, are compared in Figure 12 and Figure 13 to those directly measured (references) along these radial lines. To provide a comprehensive analysis, Figure 14 presents the reconstructions of the amplitudes of $V_{\varphi }$ and $V_{\rho }$ on the radial line at φ=π/6. It can be clearly observed that there is very good matching of the measured voltages (solid line) with the interpolated ones (crosses). Some discrepancies are observed, but only for the more peripheral zones of the scanning plane, characterized by very low voltages (lower than −50 dB). Furthermore, the interpolated voltages show a behavior which is smoother than that of the directly acquired voltages, which, on the other hand, appears rippled. This phenomenon occurs because the Dirichlet and Tschebyscheff sampling functions, and the kernel of the OSI expansions, allow one to reject the noise spatial harmonics greater than the antenna’s spatial bandwidth, thus acting as a low-pass filter. The presented reconstructions were performed by choosing *p* = *q* = 7.

**Figure 15:**
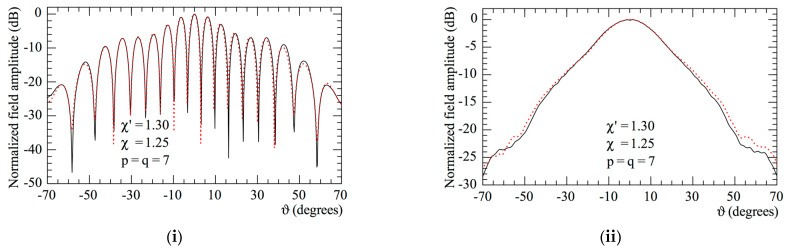
Far-field patterns. –––––, reference. ----, reconstructed from the non-redundant samples. Software co-rotation: (**i**) E-plane; (**ii**) H-plane.

**Figure 16:**
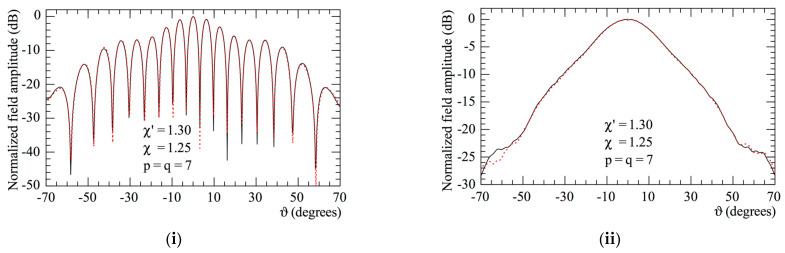
Far-field patterns. –––––, reference. ----, reconstructed from the non-redundant measurements. Hardware co-rotation: (**i**) E-plane; (**ii**) H-plane.

Finally, the use of the 2D OSI expansion followed by the application of (17) allows the efficient evaluation of the plane-rectangular data required by the standard Leach and Paris NF–FF T technique [14] from the voltages $V_{\rho }$ and $V_{\varphi }$ gathered from the spiral. The plane-rectangular sampling lattice adopted for the reconstructions lies on a square with a side length of 140 cm, inscribed in the acquisition circle, and spaced at 0.45 λ intervals. The thus-calculated FF patterns in the principal planes are compared in Figure 15 to those obtained by performing the direct measurements of the NF data at the points of the considered plane-rectangular lattice. It is evident from the reported results that the comparison is fully satisfactory in the E-plane, while that in the H-plane appears less accurate. This occurs since, unlike the open-ended circular waveguide excited by the ${\mathrm{TE}}_{11}$ mode, the far field of an open-ended rectangular waveguide excited by the fundamental mode ${\mathrm{TE}}_{10}$ exhibits only an approximately first-order azimuthal dependence [24]. For the sake of comparison, the FF reconstructions, attained by exploiting the NF spiral samples collected through the hardware co-rotation of the probe, are also presented in Figure 16. In this case, a precise reconstruction of the radiated FF pattern is achieved in both the principal planes.

Finally, the data reduction obtainable by employing the disk modeling of the AUT is summarized in Table 2. Once again, the number of NF measurements necessary for the proposed NF–FF T technique is considerably smaller than that necessary to apply the Leach and Paris transformation [14] and the classical plane-polar NF–FF T technique [25,26,27]. Moreover, the proposed NF–FF T technique compares favorably with the previous approaches [17,18] for quasi-planar AUTs from the perspective of both the reduction in the number of needed samples and computational simplicity. Note that the number of plane-rectangular samples is that needed to cover the inscribed 140 cm×140 cm square, whereas the other numbers are those required by the various techniques to cover the same scanning circle. Moreover, the semi-minor and semi-major axes of the oblate spheroid considered in the comparison are 18.6 cm and 6.0 cm in length, respectively, whilst the aperture radius of the two-bowls is 18.6 cm long and the radii of the upper and lower lateral bends are both 2.4 cm long.

## 4. Conclusions

In this article, an efficient NF–FF T technique for flat AUTs, which adopts planar spiral scanning, has been developed and thoroughly assessed both numerically and experimentally. It relies on the application of non-redundant sampling representations of the probe voltage and is obtained by assuming a flat antenna contained in a disk and exploiting the unified theory of spiral scanning for non-volumetric AUTs. An effective OSI algorithm allows the accurate evaluation of the massive amount of NF data necessary for the Leach and Paris classical transformation [14] from the non-redundant data gathered on the spiral. It must be stressed that, in the case of flat antennas, such an NF–FF T technique with planar spiral scanning allows for a further significant decrease in the acquisition time as compared to the previous methods, wherein the antenna is modeled using a two-bowl or an oblate spheroid shape. The numerical NF and FF recoveries prove the accuracy of the transformation, whereas the practical feasibility of this technique is demonstrated by the very good agreements obtained in the experimental proofs. As a concluding remark, it must be stressed that, when dealing with flat AUTs, NF–FF T techniques with planar scanning [28,29] adopting disk modeling exhibit practically the same accuracy as those based on quasi-planar antenna models [12]. On the contrary, disk AUT modeling cannot be conveniently adopted to develop NF–FF T techniques with spherical scanning for flat AUTs, since the parameter *ξ* is not analytic for *ϑ* = π/2 [1].

## Figures and Tables

**Table 1:** NF data necessary for the various NF–FF T techniques.

Plane-Rectangular NF–FF T [14]	Plane-Polar NF–FF T [25,26,27]	Planar Spiral NF–FF T [17,18] Oblate Spheroid	Planar Spiral NF–FF T [18] Double Bowl	Planar Spiral NF–FF T Disk
40,401	126,665	13,994	13,540	11,959

**Table 2:** NF data necessary for the different NF–FF T techniques.

Plane-Rectangular NF–FF T [14]	Plane-Polar NF–FF T [25,26,27]	Planar Spiral NF–FF T [17,18] Oblate Spheroid	Planar Spiral NF–FF T [18] Double Bowl	Planar Spiral NF–FF T Disk
10,816	36,177	1812	1780	1665

## Data Availability

Not applicable.
